# Using machine learning to impute legal status of immigrants in the National Health Interview Survey

**DOI:** 10.1016/j.mex.2022.101848

**Published:** 2022-09-08

**Authors:** Simon A. Ruhnke, Fernando A. Wilson, Jim P. Stimpson

**Affiliations:** a Berliner Institut für empirische Integrations- und Migrationsforschung/BIM, Berlin, Germany; b University of Utah, Matheson Center for Health Care Studies, Salt Lake City, UT; c Drexel University, Department of Health Management and Policy, PA, USA

**Keywords:** Machine Learning, Undocumented Immigrants, Demography, Immigrant, Population Health, United States

## Abstract

We describe a novel machine learning method of imputing legal status for immigrants using nationally representative survey data from the Survey of Income and Program Participation (SIPP) and the National Health Interview Survey (NHIS). K-nearest Neighbor (KNN) classifier and Random Forest (RF) Algorithm machine learning were described as novel imputation methods compared to established regression-based imputation. After validating the imputation methods using sensitivity, specificity, positive predictive value (PPV) and accuracy statistics, the Random Forest Algorithm was more accurate in identifying undocumented immigrants and minimized bias in both socio-demographic variables included in the imputation, and unobserved health variables relative to regression-based imputation and KNN.

• We developed a new machine learning method of imputing legal status for immigrants that can be used with nationally representative, publicly available data.
• Our findings indicate that using machine learning to impute legal status of immigrants, specifically the Random Forest Algorithm, was more accurate in identifying undocumented immigrants and minimized bias relative to other imputation methods.

## Graphical abstract

**Figure:**
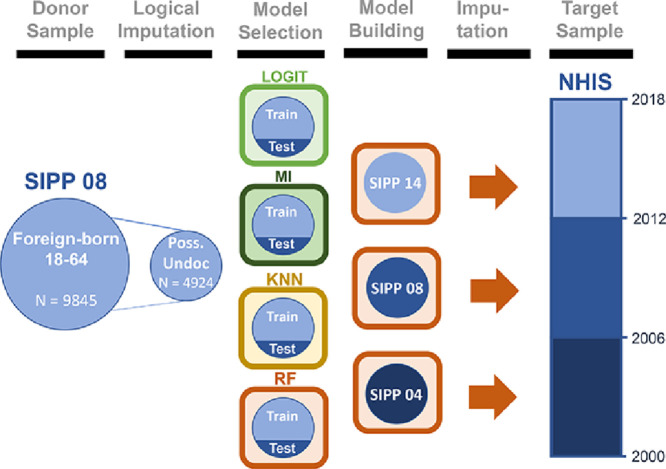

Specifications table

**Table:** 

Subject area:	Economics and Finance
More specific subject area:	*Demography, Public Health, Public Policy*
Name of your method:	*Random Forest machine learning*
Name and reference of original method:	Breiman, L. (2001). Random Forests. *Machine Learning, 45*(1), 5–32. https://doi.org/10.1023/A:1010933404324
Resource availability:	*mice and caret package in R*

## Method details

No national health survey in the U.S. captures information on the legal status of foreign-born respondents. In the absence of direct measurement, researchers studying the undocumented population have relied on proxy measures and sub-national data sources [15]. One possibility to derive quantitative evidence on the undocumented U.S. population that has been underutilized in health research is legal status imputation. In this paper we will introduce a novel approach to conduct imputation in the National Health Interview Survey (NHIS). The paper is divided into three parts. The first part provides an overview of legal status imputation methods and challenges. Next, we present a novel machine learning-based imputation approach and evaluate the performance under the sub-optimal conditions imposed by the available national health data. We accomplished this by running multiple simulations in the Survey of Income and Program Participation (SIPP). Finally, we demonstrate how the machine learning method can be applied to the NHIS and present data from this imputation on the socio-demographic composition of the undocumented population.

## Imputation methods for legal status

Rather than only using information that is given in a survey such as Green Card status to derive a measure of legal status, imputation approaches use information about the undocumented population that is external to the “target sample” to predict respondents’ legal status. This external information typically takes the form of a “donor sample”, which includes either a direct or a reliable proxy measure of legal status but lacks the size or variables of interest that are included in the target sample [13]. Rather than explicitly matching observations in the donor and target samples, which is usually not possible as respondents likely differ between the two and are anonymized, the imputation methods described in this paper predict which respondents in the target sample are most likely undocumented, based on the population characteristics derived from the donor sample.

This approach to legal status imputation allows researchers more freedom to choose a target sample that is best suited to their research question and allows for inference at a national level, but this freedom comes at a cost. Even the most sophisticated imputation approach will lack the accuracy of a good proxy or direct measure of legal status. If respondents incorrectly classified as undocumented differ systematically from the truly undocumented, legal status imputation increases the risk of introducing bias into subsequent analyses. As Van Hook and colleagues argue, this risk is particularly high if the donor sample does not include the outcome variable of interest (joint observation condition) or the donor and target sample are not derived from the same universe (same-universe condition) [13]. In our case, the outcome of interest was the muti-dimensional health status of the U.S. undocumented population. The non-existence of a national health survey that captures respondents’ legal status makes the violation of at least one of these conditions inevitable. Therefore, we chose an imputation approach that minimized the risk of introducing systematic bias and that could lead to incorrect estimates of the health of the undocumented population.

This risk of bias and the computational challenges have likely contributed to the limited use of legal status imputation in health research. Demographers on the other hand have long used imputation methods to derive information about the socio-demographic characteristics of the undocumented population. The most commonly cited source for information on size and make-up of the undocumented population in the U.S. is the Pew Research Center, which uses information from a number of public and administrative datasets (i.e. multiple donor samples) to impute legal status in their target sample: the American Community Survey (ACS) and the Current Population Survey (CPS) (J. [9]). Similarly, the Migration Policy Institute also uses the ACS as the target sample of their analysis of the U.S. undocumented population and uses the SIPP as the donor sample for their legal status imputation [6]. In a rare example of legal status imputation in health research, Wilson et. al (2020) used the Los Angles Family and Neighborhood Survey (LAFANS) as the donor sample to impute legal status in the target sample: The Medical Expenditure Panels Survey (MEPS).

Building on well-established methods used in the field of demography, this paper will explore a new method of legal status imputation for the health of the U.S. undocumented population. NHIS is the nation's largest health survey, making it an ideal target sample for health focused legal status imputation. NHIS provides the size and scope necessary to study the diverse yet small population of undocumented immigrants by interviewing roughly 35,000 households every year using a nation-wide stratified sampling strategy.

We focus on the risk of bias introduced by the violation of the joint observation condition. To date, no national survey elicits both legal status as well as detailed information on health outcomes and healthcare access. The use of legal status imputation for health research will inevitably violate the joint observation condition. Therefore, it is critical to identify an imputation method that minimizes bias under the suboptimal conditions imposed by publicly available data.

Evaluating the quality of an imputation method requires a data source capable of measuring an immigrant's true legal status. The second wave of the 2008 Survey of Income and Program Participation (SIPP 2008) provides a commonly used proxy for adult immigrants legal status by asking whether foreign-born respondents entered the U.S. as Permanent Legal Residents (LPR) and whether their status has since been adjusted to LPR [13]. Following these common practices, any non-citizen SIPP respondent entering the U.S. after 1981 without having or since adjusting to LPR status and without other indicators of legal status (see Logical Imputation below) will be treated as a truly undocumented immigrant for the purpose of this study. This binary legal status indicator will function as the target classification variable of the imputation approaches tested in this paper.

We focused on three factors to evaluate which imputation approach is best suited for imputing legal status in the NHIS. First, we evaluated the imputation method's ability to accurately assign undocumented status. Second, we evaluated the ability of the method to characterize the socio-demographic profile of the undocumented population on which the imputation is based. Third, we evaluated the method's ability to accurately assess the relationship between legal status and health-related characteristics that are not included in the donor sample, which simulates the violation of the joint-observation condition.

Borrowing from machine learning practices, the accuracy of legal status classification (other than for logical imputation) is evaluated using performance metrics based on cross-validation of the imputed and truly undocumented survey respondents, including the probability of a truly undocumented respondent being classified as undocumented (sensitivity), the probability of a truly documented individual being falsely assigned undocumented status (specificity), the probability that a respondent classified as undocumented is actually undocumented (Positive Predictive Value), and the overall percentage of cases being correctly classified (accuracy).

In a second step, we investigated whether the imputation methods lead to bias in estimating the relationship between undocumented status and health-related variables in the target sample. To capture both the domain of individual health, as well as healthcare access, we measured self-rated health (poor or fair) and private health insurance status as binary indicators. We tested for bias by calculating Pearson's correlation coefficients between undocumented status and the two binary health variables. Self-rated health is only asked in the fourth wave of the 2008 SIPP; therefore the correlation analysis is restricted to those foreign-born adult individuals that responded to both the second and fourth wave of the SIPP (N=7998).

### Logical imputation

Logical imputation is arguably the simplest legal status imputation method applied in survey research, as it does not require the use of a secondary “donor” sample. Instead, the external information used to assign legal status is a list of individual characteristics that are mutually exclusive with undocumented status. In the specification of the logical imputation approach used here, this list includes citizenship, Medicare coverage, veterans and active-duty military status, and receipt of public assistance, supplemental or social security income. Any survey respondent reporting one or more of these characteristics was logically determined to be documented. The residual, those respondents who cannot be logically determined to be documented based on their survey responses, were classified as undocumented.

The main drawback of this approach is that many truly documented individuals will remain in the undocumented sample. In our case only 33.4% of individuals (N=1672) of those classified as undocumented by logical imputation (N=4924) were truly undocumented. The selective inclusion of many documented immigrants in the undocumented group can lead to misleading conclusions about the relationship between undocumented status and health outcomes. As the results in Table 2 illustrate, logical imputation leads to an overestimate of the negative relationship between undocumented status and both poor/fair self-rated health and private health insurance, relative to the true relationship observed in the SIPP.

The number of documented immigrants falsely assigned undocumented status could be decreased by employing additional exclusion criteria such as employment in a federally licensed occupation or Medicaid coverage. Any expansion the strictly logical criteria carries with it the risk of systematic bias. Borjas and Cassidy [1] for instance determined individuals that reported being covered under Medicaid as definitively documented. Rather than a logical certainty, the association between legal status and Medicaid receipt was strongly correlated because a small number of undocumented immigrants reported Medicaid coverage in several surveys. These individuals might receive Medicaid coverage through state-level provisions that cover pregnant women, mothers, and children or misreport their coverage status due to confusion (e.g., due to previous “Emergency Medicaid” coverage) or fear of disclosing their legal status. The strict exclusion of these individuals can in turn result in misleading conclusions due to the substantial correlation between Medicaid coverage and other socio-economic indicators, most notably, poverty [12].

While the low specificity of the cautious logical imputation employed should deter its use on its own, limiting the logical imputation to logical exclusion criteria maximizes sensitivity, i.e., the excluded population contains no truly undocumented immigrants. Logical imputation can thus be employed to reduce the foreign-born sample prior to applying further imputation methods without losing any truly undocumented observations. For the remainder of this paper, we used this two-step approach.

### Logistic regression imputation

One way to improve on the results of the Logical Imputation approach is by using statistical methods to identify members of the “possibly undocumented” group that are likely undocumented based on their socio-demographic characteristics. The common statistical method used to facilitate this prediction is logistic regression modeling, which can be applied in either a single or multiple imputation framework. We will first consider the simpler Single Logistic Imputation (see [11] for an example).

Establishing a relationship between respondents’ socio-demographic characteristics and their probability of being undocumented requires as “donor sample” that includes both socio-demographic variables as well as an indicator of legal status in the form of a direct or reliable proxy measure. The information gained from the “donor sample” can then be used to predict undocumented status in the “target sample”. To simulate this donor-target relationship within the SIPP, we follow common machine learning practices by randomly splitting the SIPP into a training and a test sample. Our test sample consists of 20% of the initial SIPP sample and the undocumented identifier is muted, hence simulating a “target sample” that is missing this information. The remaining 80% of the SIPP that make up the training sample remain unchanged, representing the “donor sample” with the full set of information. As described above, both samples are subset to include only the “possibly undocumented” identified by the Logical Imputation. The procedure is repeated for ten different random splits of training and test data and the results presented in Tables 1 and 2 averaged across the ten iterations to ensure that the results are not driven by any one random split.

**Table 1:** Average model performance metrics of logistic, K-Nearest neighbor and random forest using bootstrapped cross-validation.

	Logit	KNN	RF
Sensitivity	0.68995	0.68478	0.71828
Specificity	0.57205	0.57092	0.61948
PPV	0.34462	0.29311	0.43952
Accuracy	0.86738	0.88600	0.86152

**Table 2:** Average correlations between (Imputed) legal status and health variables using bootstrapped cross-validation.

Correlation between:	Legal Status & Private Health Insurance	Legal Status & Poor/Fair Health
	Pearson's Cor. Coef. (95% CI)	Pearson's Cor. Coef. (95% CI)
True relationship in full SIPP	-0.2245	-0.0375
(N = 7998)	(-0.2431; -0.2056)	(-0.0594; -0.02)
TRUE Relationship in Test-SIPP	-0.16823	-0.01478
(N = 984)	(-0.2283; -0.1316)	(-0.0851; 0.0557)
Logical	-0.276	-0.0611
	-0.2942; -0.2577	-0.0829; -0.04
Logit	-0.27218	-0.0495
	(-0.329; -0.2134)	(-0.1195; 0.021)
MI	-0.14931	-0.01666
	(-0.2463; -0.0494)	(-0.1188; 0.0859)
KNN	-0.20708	-0.02859
	(-0.2661; -0.1465)	(-0.0988; 0.0419)
RF	-0.17275	-0.04385
	(-0.2327; -0.1115)	(-0.1139; 0.0266)

The predictors used to build the logistic regression model are years lived in the U.S., educational attainment, poverty status, region of birth, marital status, difficulties speaking English, Medicaid coverage, household size, spousal citizenship, age, number of children, employment status, race and Hispanic ethnicity. Rather than imputing (using the Census provided imputations) or case-wise deleting missing values, we coded them as an additional level for categorical predictors because non-response, specifically to immigration-related questions cannot be expected to be missing-at-random. An alternative specification that includes self-rated health as a predictor was considered and results for this alternative specification are reported in Tables 3 and 4. We opted for the final predictor set presented here because self-rated health is only available for those respondents retained in the SIPP's fourth wave, and the model performance does not indicate substantial improvements in prediction performance warranting this loss of observations.

**Table 3:** Average model performance metrics of logical, single logistic, K-Nearest neighbor and random forest using bootstrapped cross-validation, alternative specification including self-rated health as a predictor.

	Logit	KNN	RF
Sensitivity	0.69401	0.68589	0.72328
Specificity	0.58148	0.56923	0.62773
PPV	0.35240	0.30720	0.45389
Accuracy	0.86954	0.88048	0.86169

**Table 4:** Average Correlations Between (Imputed) Legal Status and Health Variables using bootstrapped Cross-Validation, alternative specification including self-rated health as a predictor.

Correlation between	Legal Status & Private Health Insurance	Legal Status & Poor/Fair Health
	Pearson's Cor. Coef. (95% CI)	Pearson's Cor. Coef. (95% CI)
True relationship in full SIPP	-0.2245	-0.0375
(N = 9845)	(-0.2431; -0.2056)	(-0.0594; -0.02)
TRUE Relationship in Test-SIPP	-0.16823	-0.01478
(N = 984)	(-0.2283; -0.1316)	(-0.0851; 0.0557)
Logit	-0.26579	-0.0369
	(-0.3229; -0.2067)	(-0.107; 0.0336)
MI	-0.1441	-0.02003
	(-0.2378; -0.0477)	(-0.1237; 0.0841)
KNN	-0.19708	-0.04334
	(-0.2564; -0.1363)	(-0.1134; 0.0272)
RF	-0.17478	-0.03421
	(-0.2347; -0.1135)	(-0.1043; 0.0363)

Model coefficients were derived from running the logistic regression on the training sample. After predicting the probability of being undocumented among respondents in the test sample, all those with a predicted probability greater than 50% are determined to be undocumented. Model performance indicators are reported in Table 1. Unlike the Logical Imputation, the Logistic Imputation leads to some undocumented immigrants being falsely assigned documented status, resulting in a sensitivity of 0.69. With a PPV of 0.35, it only presents a minor improvement in the share of truly undocumented individuals in the imputed undocumented group over the Logical Imputation. As the results in Table 2 illustrate, Logistic Imputation also results in a significant overestimation of the negative relationship of undocumented status and health insurance coverage relative to the true relationship in the test sample (-0.27 vs. -0.17). Both the imputed correlation between undocumented status and self-rated health and the true correlation in the test SIPP were insignificant.

The main benefit of using a Multiple rather than a Single Imputation framework is the ability to capture the uncertainty inherent in any attempt to predict legal status in a sample that lacks this information. In practice, any analysis using legal status based on multiple imputations will yield higher Standard Errors to account for this uncertainty [13]. In extensive testing, Van Hook et al [13] also show that Multiple Imputation (MI) based on logistic regression yields unbiased estimates of the relationship between legal status and insurance coverage. But this result only holds if the joint observation condition is met, i.e., if legal and health insurance status are both observed in the donor sample. As described above, research on the health of the U.S. undocumented population must inevitably violate this condition when using any form of cross-survey imputation. Thus, we must evaluate whether MI can reduce the resulting bias relative to the Single Imputation approach tested above, even under the sub-optimal conditions imposed by publicly available data.

Following common practice, the MI approach builds on Logistic Imputation using chained equations facilitated by the *mice* package in R [4]. We used the same logistic specification as outlined above but instead of predicting legal status once in the test data, the MI approach creates ten separate test datasets, that are all equal except for differences in the respondent's imputed legal status. All subsequent analysis is then performed in all 10 datasets separately and results are pooled to account for the uncertainty in imputing legal status.

Unlike Single Imputation, MI treats cross-survey legal status imputation as missing data rather than a prediction problem. Because of this philosophical difference, MI is not designed to provide a definitive classification for each observation. Instead, it has been designed to impute missing values in datasets while retaining the statistical relationship between all variables in the model. This poses several practical constraints with regards to the ultimate analysis in the target sample following legal status imputation. Because MI doesn't assign a definitive legal status, the results cannot be easily combined with additional variables in the target sample to use in the final analysis. All variables used in the final analysis must instead be included in the imputation itself, regardless of their predictive power.

Moreover, cross-validation and socio-demographic summary statistics of the imputed undocumented population are not meaningful measures when evaluating the MI approach. Instead, we relied on the Pearson correlation coefficient between legal status and the health variable to assess the ability of MI to reduce bias. The results in Table 2 show that MI leads to a small positive bias in the relationship between imputed legal and private health insurance status and reproduces the insignificant correlation between undocumented status and self-rated health found in the test sample.

### Machine learning imputation

Unlike traditional regression models, non-parametric machine learning algorithms do not require any prior assumptions about the functional form that is underlying the relationship between socio-demographic predictors and undocumented status. With this increased flexibility, non-parametric models can account for more complex relationships and can reduce the bias observed in Logistic Regression Imputation.

One of the most popular non-parametric machine learning classification algorithms is the K-nearest Neighbor (KNN) classifier. The basic idea underlying the KNN approach is to identify the k observations that are most similar to the observation for which classification is required. Whichever class the majority of these “nearest neighbors” belong to is assigned to the observation in question. Similarity or “nearness” between different observations is established via Euclidean Distance in an n-dimensional space, with n being equal to the number of selected predictors [7]. To account for differences in units of measurement and possible maximum and minimum values, predictors are typically normalized.

To compare the performance of the KNN algorithm to the Logistic Imputation approach we used the same test and training samples as above, as well as the same 15 predictors. The optimal value for k is determined to be 31 based on repeated 10-fold cross validations using the caret package in R (Max [8]). As the results in Table 1 show, the KNN Imputation results in a lower sensitivity and slightly higher specificity than the Logistic Regression Imputation, resulting in a lower PPV of 0.29 and a slightly higher accuracy of 88.6. Like the Single Logit Imputation, KNN overestimates the negative relationship of legal status and private health insurance, but with a correlation coefficient of -0.207, it does so to a smaller extent. The correlation coefficient between KNN-imputed legal status and self-rated health also shows a slight negative bias relative to the true relationship in the test sample but remains insignificant. Overall, the KNN Imputation only offers a minor improvement over the Logistic Regression imputation in terms of bias, at the expense of lower sensitivity in identifying undocumented respondents.

Another non-parametric machine learning algorithm is the Random Forest (RF) Algorithm [2]. It builds on the concept of the decision tree, where each node of the branch represents a predictor, and each branch ends in an assignment to a class group. The RF Algorithm grows a large ensemble of decision trees, each based on a random subsample of both the training sample (drawn with replacement) and the predictors (drawn-without replacement). The algorithm chooses node-splits that maximize homogeneity in the resulting split groups. RF is referred to as an ensemble method, as each tree is grown independently and produces a prediction for each observation in the test sample. These “votes'' are then aggregated across trees, leading to the final categorization of each observation based on the majority vote. This approach reduces the risk of overfitting the model that often occurs in simple decision tree models, i.e. on average the RF algorithm performs better in unknown data than comparable models [3]. With the large number of interactions between predictors introduced by the tree design, the RF algorithm also has the potential to account for more complex relationships between socio-demographic characteristics and legal status.

Like the KNN Imputation, we used the same training data for the using 10-fold repeated cross validations for the RF algorithm. The results based on a forest of 500 trees shows 12 to be the optimal number of predictors randomly drawn for each tree from the same predictors as described above. The results of running the test data through the tuned model are reported in Table 1. The RF outperforms both logistic and KNN imputation in terms of sensitivity, specificity and yields the highest PPV among the three with a value of 0.44. With a correlation coefficient between RF-imputed undocumented status and private health insurance coverage of -0.173, the RF had the best reproduction of the true relationship in the test data among all tested approaches (Table 2). Like the other approaches, and in line with the results in the test data, RF produces an insignificant correlation between undocumented status and self-rated health.

In summary, non-parametric Machine Learning approaches provide a viable alternative to existing strategies in legal status imputation [14]. Specially the Random Forest Algorithm shows superior performance compared to traditional approaches as it is more accurate in identifying undocumented immigrants and minimizes bias in both socio-demographic variables included in the imputation, as well as in unobserved health variables relative to regression-based imputation.

## Application to the National Health Interview Survey

Having identified the RF approach as the best performing imputation method under the suboptimal conditions imposed by the availability of suitable national health survey data, we applied it to the NHIS. The NHIS is a stratified random sample of the non-nationalized U.S. population conducted by the National Center for Health Statistics (NCHS). While the basic socio-demographic information needed for legal status imputation is available for all household members, detailed health information is only captured for one adult respondent per household. We will thus restrict the imputation to this Adult Sample.

Despite the large, nationally representative sample of the NHIS, the small share of undocumented immigrants in the U.S. can still yield small cell-sizes each year when stratifying the final analysis by factors such as years lived in the U.S., region of origin or healthcare access status. Samples sizes can be increased by pooling multiple years of the NHIS. Moreover, the composition of the U.S. undocumented population has changed markedly over time, as is evident in the descriptive statistics for the 2004, 2008 and 2014 SIPP presented in Table 5, as well as in the results presented by the Pew Research Center [10]).

**Table 5:** Socio-economic characteristics in the SIPP 2004, SIPP 2008, SIPP 2014, NHIS (2000-2018) using the Random Forest algorithm to impute documentation status.

	SIPP 2004			SIPP 2008			SIPP 2014			NHIS 2000-2018		
	US-born	Documented	Undocumented	US-born	Documented	Undocumented	US-born	Documented	Undocumented	US-born	Documented	Undocumented
	(%)	(%)	(%)	(%)	(%)	(%)	(%)	(%)	(%)	(%)	(%)	(%)
**Age**	40.35	40.31	32.72	40.93	41.22	33.47	41.11	42.75	36.15	40.79	41.05	35.2
**Marital Status**												
Married	55.22	63.75	60.22	52.91	63.47	53.45	48.86	64.83	53.77	52.92	64.53	60.90
Widowed	1.96	1.67	0.51	1.86	1.91	0.97	1.82	1.66	0.87	1.91	1.79	0.90
Divorced	11.93	8.07	2.45	11.82	8.14	3.86	12.03	8.92	4.95	11.80	7.73	4.38
Separated	2.12	3.40	3.03	1.95	2.99	3.29	2.20	2.76	3.48	2.32	3.40	3.51
Never Married	28.37	22.43	33.32	30.84	22.50	37.08	34.79	21.38	35.84	30.76	22.18	29.97
Missing	0.40	0.68	0.46	0.62	0.98	1.34	0.30	0.45	1.08	0.30	0.38	0.33
**Race**												
White	83.33	67.55	78.28	82.90	65.16	78.06	79.01	44.38	44.75	83.47	62.84	69.96
Black	12.59	9.78	7.22	12.69	11.42	7.93	13.49	9.17	5.79	13.48	10.27	7.06
Asian	0.94	19.25	13.28	1.19	20.05	10.97	1.33	25.25	16.54	1.42	23.29	19.73
Other	3.15	3.42	1.21	3.22	3.37	3.04	3.26	2.71	0.97	1.64	3.59	3.24
Missing	0	0	0	0	0	0	2.30	15.67	28.85	0	0	0
**Hispanic**												
No	92.49	57.31	38.50	91.41	58.48	31.67	90.58	56.80	32.32	92.42	51.93	35.26
Yes	7.51	42.69	61.50	8.59	41.52	68.33	9.34	43.14	67.55	7.58	48.07	64.74
**Employed**												
No	24.32	26.35	31.68	27.03	28.86	34.95	30.47	29.90	33.51	27.41	27.46	32.79
Yes	75.68	73.65	68.32	72.97	71.14	65.05	69.53	70.10	66.49	72.45	72.36	66.86
**Educational Attainment**												
Never Attended	0.14	1.33	2.05	0.09	0.98	1.96	0.06	0.83	1.36	0.15	1.20	1.61
1-6 Grade	0.51	11.46	22.43	0.39	9.25	18.32	0.17	6.68	17.87	0.40	9.70	17.12
7-12 Grade	8.34	14.90	20.92	6.98	11.32	19.98	7.97	12.41	20.26	9.42	15.26	21.60
Highschool	26.76	20.25	20.05	26.23	23.92	28.42	28.96	23.11	21.37	27.45	21.34	20.48
Some College	38.18	25.67	13.07	38.03	26.13	12.49	32.30	22.25	13.76	33.36	21.38	14.34
Undergrad	17.10	15.32	11.08	18.39	17.19	8.49	19.62	20.26	11.82	19.28	17.94	13.28
Graduate	7.33	7.92	6.63	8.23	8.24	5.17	10.10	11.67	10.30	9.44	11.61	9.80
Missing	1.65	3.16	3.77	1.67	2.98	5.17	0.83	2.79	3.26	0.51	1.56	1.76
**Income/Poverty Ratio**												
Below 100% FPL	11.28	14.92	23.12	13.24	17.49	32.19	15.03	16.98	29.81	11.74	17.92	26.88
Below 200% FPL	14.96	22.86	35.11	15.15	23.66	32.96	15.43	20.91	28.06	15.54	23.57	31.43
Above 200% FPL	73.76	62.23	41.78	71.62	58.85	34.85	69.53	62.11	42.14	72.72	58.51	41.69
**Region of Birth**												
USA	100	0	0	100	0	0	100	0	0	100	0	0
Central/South America	0.00	56.73	74.92	0.00	51.86	79.35	0.00	51.50	70.72	0.00	53.81	67.73
Europe	0.00	13.08	4.55	0.00	12.15	3.44	0.00	12.51	5.33	0.00	12.36	5.42
Africa	0.00	3.19	4.26	0.00	4.08	3.22	0.00	4.17	3.98	0.00	4.34	3.36
Asia	0.00	23.84	14.77	0.00	24.25	11.97	0.00	31.06	18.77	0.00	25.87	20.30
Other	0.00	3.17	1.49	0.00	7.65	2.03	0.00	0.76	1.20	0.00	2.95	2.17
**Years in the US**												
< 5 years	0.00	33.13	43.87	0.00	16.98	33.31	0.00	6.01	15.34	0.00	10.38	27.25
5-10 years	0.00	14.22	38.21	0.00	23.28	32.87	0.00	10.36	18.76	0.00	12.52	24.28
10-15 years	0.00	13.86	10.92	0.00	14.15	17.65	0.00	12.27	23.61	0.00	14.76	17.30
15+ years	0.00	38.79	7.00	0.00	45.60	16.17	0.00	50.44	36.15	0.00	59.59	28.67
Missing	100.00	0	0	100.00	0	0	100.00	20.93	6.01	100.00	2.75	2.50
**Difficulties Speaking English**												
No	99.24	74.22	43.94	99.31	77.04	48.73	99.84	86.13	74.29	98.89	71.12	51.17
Yes	0.76	25.78	56.06	0.69	22.96	51.27	0.16	13.82	25.71	0.92	28.66	48.56
**Number of Children**	0.77	1.05	1.18	0.74	1.03	1.2	0.73	1.18	1.28	0.78	1.17	1.14
**Household Size**	3.11	3.72	4.11	3.09	3.67	4.34	3	3.64	3.97	2.91	3.54	3.76
**Medicaid**												
No	91.71	89.48	92.19	91.61	90.13	91.95	84.64	80.56	81.78	92.38	91.48	92.47
Yes	7.38	9.08	6.67	7.12	8.06	7.45	9.23	11.83	10.46	7.13	7.98	6.70
**Spousal Citizenship**												
No	0.88	21.22	45.55	0.97	21.10	40.34	0.89	18.65	36.03	1.11	25.78	47.70
Yes	53.62	38.95	9.18	51.32	39.58	8.12	46.93	43.14	13.93	58.97	40.68	15.77
Missing	45.50	39.83	45.26	47.71	39.33	51.54	52.18	38.22	50.04	39.92	33.54	36.52
**Region of Residence**												
Northeast	18.12	22.91	18.05	17.91	20.50	12.40	17.11	22.01	16.50	17.05	21.77	16.75
Midwest	24.52	11.52	9.29	23.79	11.80	13.20	23.44	12.22	12.34	25.92	12.06	11.40
South	36.75	29.74	34.57	37.25	32.42	37.00	38.19	31.87	36.74	37.32	31.95	34.37
West	20.62	35.83	38.09	21.05	35.28	37.40	21.26	33.89	34.42	19.71	34.22	37.47
**Poor/Fair SRH**												
No	79.03	76.90	79.02	74.91	73.68	67.54	79.81	79.75	83.19	89.29	89.82	92.18
Yes	10.52	10.22	6.29	10.02	9.39	6.20	12.99	11.71	8.11	10.65	10.13	7.79
Missing	10.45	12.88	14.69	15.07	16.93	26.26	7.2	8.54	8.7	0.06	0.05	0.03

Results expressed in weighted Column Percentages.

Source: United States Census: Survey of Income and Program Participation 2004, Wave 2&3; 2008, Wave 2&4, 2014, Wave 1; National Health Interview Survey 2000-2018.

FPL: Federal Poverty Line (Household Income).

SRH: Self-Rated Health.

To account for the changing composition of the U.S. undocumented population, we grow separate RF models, following the approach presented above in the 2004, 2008 and 2014 cohorts of the SIPP and apply them to NHIS cross-sections from 2000 to 2006, 2007 to 2012 and 2013 to 2018, respectively. A limitation of this approach is the substantial change in SIPP's survey design between the years 2008 and 2014. Most notably, the question whether respondents have changed their status to “permanent” since arriving in the U.S. is dropped from the survey entirely, making the legal status proxy and thus subsequent imputation based on it less accurate than previous SIPP cohorts. This inconsistency can be addressed by either using only the 2004 and 2008 cohorts of the SIPP for imputation across all years of the NHIS, risking inaccuracy for the later years, or by dropping the later observations of the NHIS entirely, restricting the final analysis to more historic data. Alternatively, one can capture any systemic differences in the characteristics of the imputed undocumented population between years by including year fixed effects in the final statistical analysis in the NHIS thus avoiding confounding bias resulting from the different donor samples but rendering a longitudinal analysis of the NHIS data impractical.

The presented imputation approach also faces challenges from differences between the SIPP and NHIS. While ostensibly sampling randomly from the same universe, i.e., the non-institutionalized U.S. population, at roughly the same time, differences in sampling strategies between SIPP and NHIS and thus in sample selection are unavoidable, especially when surveys are conducted by different organizations, as is the case here. One organization might, for example, have more translators available, resulting in a higher probability of non-English speaking individuals being selected into the survey. There are multiple approaches to account for such differences in the probability of being sampled. One approach is to assign a propensity score to each respondent representing the probability of being selected into the SIPP, based on observable characteristics, including the predictors used in the imputation model. This propensity score is then included as an additional predictor in the imputation, thus reducing possible bias introduced into the imputation by systematic differences in the sampling probability between the SIPP and the NHIS [5]. In the specification presented here, in addition to the predictors included in the logistic regression model, individual's region of residence and occupation were added to calculate the propensity score.

The results of applying the discussed RF imputation approach to the pooled cross-section of the NHIS are presented in Table 5. The NHIS respondents defined as “documented” in the table include both those excluded from the undocumented population via logical edits, as well as the “possibly undocumented” that were excluded based on the imputation model. Consistent with previous research and SIPP data, the imputed undocumented population in the NHIS is on average younger, more likely to be Hispanic and of lower socio-economic status than their documented counterparts. The main difference between the imputed NHIS sample and the SIPP sample is a smaller proportion of undocumented immigrants originating from Central and South America and, correspondingly, a larger proportion from Asia. Future research that uses non-publicly available data with detailed information on country of origin should consider stratifying the legal status imputation by region of origin to account for this discrepancy.

### Application to other data sources and questions

The use of the multi-survey Random Forest imputation approach is not dependent on health data and could be applied to other fields and topics where quantitative data sources that identify undocumented immigrants are scarce or unavailable. Quantitative research in critical, yet under-researched dimensions of undocumented immigrants’ wellbeing in the U.S., such as discrimination, integration, or job market experience, could thus be possible using the methodology advanced in this paper.

Legal status imputation also provides an avenue for junior researchers to engage in quantitative research that concerns undocumented immigrants. This group of scientists, which includes many individuals with close ties to the communities involved, often lack access to the resources necessary to conduct primary data collection. By enabling the use of publicly available secondary data, advances in legal status imputation could thus be a means to promote diversity in research concerning the U.S. undocumented population. In such efforts to democratize data access, it remains imperative that the anonymity of survey respondents remains ensured and that imputations methods are used for scientific inquiry only.

## Ethics statements

Our work used publicly available data and does not meet the definition of human subjects research.

## CRediT authorship contribution statement

**Simon A. Ruhnke:** Conceptualization, Methodology, Formal analysis, Writing – original draft. **Fernando A. Wilson:** Methodology, Resources, Writing – review & editing, Supervision. **Jim P. Stimpson:** Writing – review & editing, Supervision.

## Declaration of Competing Interest

The authors declare that they have no known competing financial interests or personal relationships that could have appeared to influence the work reported in this paper.

## Data Availability

Publicly available data were used that are available to download from the internet for free.
